# FoxO6 and PGC-1α form a regulatory loop in myogenic cells

**DOI:** 10.1042/BSR20130031

**Published:** 2013-06-13

**Authors:** Shih Ying Chung, Wei Chieh Huang, Ching Wen Su, Kuan Wei Lee, Hsiang Cheng Chi, Cheng Tao Lin, Szu-Tah Chen, Kai Min Huang, Mu Shiun Tsai, Hui Peng Yu, Shen Liang Chen

**Affiliations:** *Department of Life Sciences, National Central University, Jhongli 32001, Taiwan; †Division of Endocrinology and Metabolism, Chang Gung Memorial Hospital, Kweishan 33333, Taoyuan, Taiwan; ‡Department of Pathology and Medical laboratory, Taiwan Landseed Hospital, Pingjen 32449, Taoyuan, Taiwan

**Keywords:** FoxO6, muscle, oxidative metabolism, *PGC-1*α, promoter, transcription, CHIP, chromatin immunoprecipitation, DM, differentiation medium, DMEM, Dulbecco’s modified Eagle’s medium, EMSA, electrophoretic mobility-shift assay, FCS, fetal calf serum, FoxO, forkhead box O, Gapdh, glyceraldehydes-3-phosphate dehydrogenase, GC, gastrocnemius, GM, growth medium, HEK-293T, HEK-293 cells expressing the large T-antigen of SV40, HRP, horseradish peroxidase, MBP, maltose-binding protein, MRFs, myogenic regulatory factors, PBST, PBS containing 0.5% Tween 20, PGC-1α, peroxisome-proliferator-activated receptor γ co-activator 1α, PI3k, phosphoinositide 3-kinase, qRT-PCR, quantitative reverse transcription PCR, T2D, type 2 diabetes

## Abstract

Transcription factors of the FoxO (forkhead box O) family regulate a wide range of cellular physiological processes, including metabolic adaptation and myogenic differentiation. The transcriptional activity of most FoxO members is inhibitory to myogenic differentiation and overexpression of *FoxO1* inhibits the development of oxidative type I fibres *in vivo*. In this study, we found that *FoxO6*, the last discovered *FoxO* family member, is expressed ubiquitously in various tissues but with higher expression levels in oxidative tissues, such as brain and oxidative muscles. Both the expression level and promoter activity of *FoxO6* were found to be enhanced by PGC-1α (peroxisome-proliferator-activated receptor γ co-activator 1α), thus explained its enriched expression in oxidative tissues. We further demonstrated that FoxO6 represses the expression of *PGC-1*α via direct binding to an upstream A/T-rich element (**AA**G**A**T**A**T**CAAAACA**,−2228–2215) in the *PGC-1*α promoter. Oxidative low-intensity exercise induced *PGC-1*α but reduced *FoxO6* expression levels in hind leg muscles, and the binding of FoxO6 to *PGC-1*α promoter was also prevented by exercise. As *FoxO6* promoter can be co-activated by PGC-1α and its promoter in turn can be repressed by FoxO6, it suggests that FoxO6 and PGC-1α form a regulatory loop for setting oxidative metabolism level in the skeletal muscle, which can be entrained by exercise.

## INTRODUCTION

The transcription factors of the FoxO (forkhead box O) family, including FoxO1 (FKHR), FoxO3 (FKHRL1), FoxO4 (AFX) and FoxO6, have been discovered to play important roles in a diverse cellular physiological functions, including glucose metabolism, stress response, differentiation, cell cycle regulation and apoptosis [1,2]. Their binding to the *cis*-response elements (consensus sequence: **TTGTTTAC**) of target genes is mediated by the forkhead-box DNA-binding domains located at the central region of these factors. In addition to direct binding to DNA, FoxOs can also function as a transcriptional co-regulator of other DNA-binding transcription factors, such as nuclear receptors and HNF4 (hepatocyte nuclear factor 4), through protein–protein interaction [3]. Furthermore, their transcriptional activity is critically regulated by signal transduction pathways-mediated post-translational modification [4]. For instance, insulin-activated PI3k (phosphoinositide 3-kinase)–Akt pathway phosphorylates FoxO1 on T24, S256 and S319 residues and this modification renders FoxO1 to be shuttled out of nucleus [5,6]. These three Akt-targeted sites, named as T1, S1 and S2, are conserved from *Daf16* in *Caenorhabditis elegan*s to its orthologues in mammals [7].

Members of the *FoxO* family are highly expressed in primary myoblasts. The expression level of *FoxO1–4* remains largely unchanged during the terminal myogenic differentiation. However, the levels of their phosphorylation and nuclear localization are significantly increased in well-differentiated myotubes [8,9]. These observations are contradictory to what observed in other cell lineages where phosphorylated FoxOs tends to be shuttled out of nucleus, and it suggests that FoxOs might behave differently in myogenic cells. Recent studies have shown that ectopic overexpression of constitutively active *FoxO* mutants in myoblasts prevents/retards myogenic differentiation or induces atrophy [9–11]. Transgenic mice overexpressing *FoxO1* show reduced muscle mass, down-regulated type I fibre genes and impaired glycaemic control [12]. It has been demonstrated that FoxOs induce muscular atrophy through activating the transcription of muscle-specific ubiquitin ligases *Atrogin-1* and *MuRF1* as well as a novel ubiquitin-binding protein called as ZNF216 [10,11,13]. Unfortunately, the direct targets of FoxOs in the genetic axis of myogenesis have not yet been identified to date.

The last discovered member, FoxO6, of this family was identified by screening novel forkhead transcription factors expressed in the ventral midbrain [14]. Its high expression in the central nervous system and dorsal root ganglion was later confirmed [15]. Outside the nervous system, it is also expressed in the thymus, cortex of kidney and limb buds [14]. Its expression in the somites or having any role in trunk muscle development has not been reported yet. One distinguishing character of FoxO6 is its constant/high nuclear localization upon growth factors stimulation due to lacking of a phosphorylation motif (S316) conserved in other FoxOs. Nevertheless, its transcriptional activity is regulated by the modification of Thr^26^ and Ser^184^, two conserved phosphrylation sites, but which is independent of nucleocytoplasmic shuttling [16].

FoxOs have been reported to regulate oxidative metabolism by directly activating the expression of *PGC-1*α (peroxisome-proliferation-activated receptor γ co-activator 1α) [17], which is a versatile transcriptional co-activator playing critical roles in the biogenesis of mitochondria, gluconeogenesis and fatty acid oxidation [18,19]. In muscle, *PGC-1*α is preferentially expressed in slow-twitch fibres and its enhanced expression can convert fast-twitch fibres into slow-twitch fibres *in vivo* [20]. The activation of slow-twitch muscle-specific genes by PGC-1α is mediated through the binding of Mef2 transcription factors on their upstream enhancer sites. Mef2 proteins also activate the transcription of *PGC-1*α gene and thus establish a positive feedback loop [21].

In this study, we examined the expression pattern of *FoxO6* in various tissues and during myogenic differentiation at both mRNA and protein levels. We found that *FoxO6* is expressed ubiquitously but with higher expression levels in oxidative tissues, such as brain and oxidative muscles. During myogenesis, *FoxO6* expression is only activated in differentiated myotubes regardless of fibre types. We further demonstrated that FoxO6 represses the expression of *PGC-1*α via direct binding to an upstream A/T-rich element (**AA**G**A**T**A**T**CAAAACA**,−2286 to −2273) and this repression can be released by exercise. Co-activation of *FoxO6* promoter by PGC-1α was also observed, which indicates that FoxO6 and PGC-1 form a regulatory loop for oxidative metabolism. This study has identified the second *FoxO6* target gene outside the nervous system and suggests the possible implication of FoxO6 in regulating oxidative metabolism by repressing *PGC-1*α expression.

## MATERIALS AND METHODS

### Plasmids

The reporter construct containing the full length (−3170 to +101) *PGC-1*α promoter has been described before [22], but with the numbering system changed to the latest identified transcription initiation site (accession no.: NC_000071.6/GI:372099105). The deletion mutants of this promoter were constructed by digesting the full-length clone with either EcoRV or PstI in conjunction with KpnI to create reporters containing −2222 to +101 and −762 to +22 regions of this promoter. Reporters containing other regions of the *PGC-1*α promoter were created by inserting *pfu* DNA polymerase-amplified PCR products into the SmaI site of pGL2-tk-enhancer vector (Promega). All PCR products were sequenced to confirm their sequence integrity. The expression vector pMSCV–FoxO6 was created by inserting *FoxO6* coding sequence released from pEGFP–FoxO6 vector (a generous gift from Dr Marten P. Smidt; Department of Pharmacology and Anatomy, University Medical Center, Ultrecht) with SalI/BamHI (blunt) into the *Xho*I site of pMSCV vector.pCDNA3–FoxO6–MCS was created by inserting *FoxO6* CDS released from pEGFP–FoxO6 vector with HindIII/BamHI into the same sites in pCDNA3 vector to generate a chimaeric gene containing FoxO6 CDS and the multiple cloning sites (BamHI to XbaI, 78 bp) of the vector. Similarly, *FoxO6* CDS was amplified with primers containing Flag-tag and inserted into the XhoI site of the pPyCAG-IP vector to create C-terminal Flag-tagged *FoxO6* expression vector that allows stable selection with puromycin. The CDS (559 amino acids) of FoxO6 was also amplified from C2C12 cDNA and cloned into the EcoRV site of pCDNA3 vector to generate pCDNA3–FoxO6. The least conserved region (mRNA+685 to +1476, amino acid 229–492) of *FoxO6* was inserted into the BamHI/EcoRI sites of modified pET-32a vector, in which the 3′-His-tag was made in-frame by blunting the NotI/XhoI sites, to generate FoxO6 (229–492) protein with His-tag on both ends. *FoxO6* promoter (−2977 to +299) was amplified from mouse genomic DNA by PCR and inserted into the HindIII and XhoI sites of the pGL3-basic vector.

### Cell culture and transient promoter assay

Proliferating C2C12 myoblasts were kept in GM (growth medium) containing DMEM (Dulbecco's modified Eagle's medium) supplemented with 20% (v/v) FCS (fetal calf serum). Myogenic differentiation was induced by replacing the medium with DM (differentiation medium) containing DMEM supplemented with 2% (v/v) horse serum. For transient transfection, *PGC-1*α promoter-driven reporter vector (0.67 μg) and pMSCV–FoxO6 (0.33 μg) were mixed together in 1× Hepes buffer (20 mM Hepes at pH 7.0, 187 mM NaCl, 5 mM KCl, 0.7 mM Na_2_HPO_4_ and 5.5 mM dextrose) in 1.5 ml test tubes and then liposome (Lipofectamin, Invitrogen) in 1× Hepes buffer ware added to the DNA mixture and incubated at room temperature (25°C) for 10–15 min to allow DNA and liposome complex to form. Aliquots (1 ml) of culture medium were added to each tube and mixed with several inverting. Medium containing the DNA/liposome complex in each tube was transferred to cells grown in a 12-well Petri dish and the transfection was allowed to proceed overnight before the medium was replaced by fresh DM containing either 2% (v/v) horse serum or GM containing 20% (v/v) FCS. Cells were harvested and assayed for luciferase activity 48 h after transfection in a Clarity 2 luminometer (BioTEK; Winooski, VM). All experiments were performed in triplicates and repeated at least three times.

### Stable cloning of FoxO6 overexpressed C2C12 myoblasts

The stable cloning of C2C12 myoblasts has been described before [23]. Briefly, C2C12 grown on a single well of a 6-well Petri dish were transfected with pPyCAG-IP–FoxO6 for overnight and replaced with fresh GM. After 48 h, myoblasts were trypsinzed and plated into a 10 cm dish and started selection with puromycin (3 μg/ml). Antibiotic selection was continued for 2 weeks before multiple colonies shown up on the dish. Then, cells were trypsinzed to mix all the colonies and kept in the same concentration of puromycin in the following passages. The expression of *FoxO6* was confirmed with RT-PCR.

### qRT-PCR (quantitative reverse transcription PCR)

The protocol for qRT-PCR has been described previously [24]. Briefly, total RNA was extracted from C2C12 cells using TRIZOLE kit, then, the first strand of cDNA was synthesized using the Superscript III kit according to the supplier's instruction. Quantitative real-time PCR was performed in a 20 μl reaction mixture containing 5 μM forward/reverse primers, 1× SYBR Green reaction mix (Applied Biosystems) and various amounts (equivalent to about 25, 50 and 100 ng of the total RNA) of templates. A tube containing equivalent amount of total RNA was used as reverse transcriptase control and amplified in the same PCR reaction. Only samples shown free of genomic contamination were further analysed. *Gapdh* (glyceraldehydes-3-phosphate dehydrogenase) was used as internal control amplified in the same PCR assay. The primer sets used were listed in Table 1. All reactions were performed in an ABI 7300 sequence detection system.

**Table 1:** The sequences and amplicon sizes of the primer sets used in this study

Gene	Amplicon size (bp)	Forward primer	Reverse primer	Purpose
*Gapdh*	190	5′-CCTCTGGAAAGCTGTGGCGT-3′	5′-TTGGCAGGTTTCTCCAGGCG-3′	qRT-PCR
*PGC-1*α	166	5′-GAGCGCCGTGTGATTTACGT-3′	5′-GCGAAAGCGTCACAGGTGTA-3′	qRT-PCR
*FoxO6*	172	5′-CTCACGCTCTCGCAGATCTA-3′	5′-GCATCCACCATGAACTCTTGC-3′	qRT-PCR
*PGC-1*α	252	5′-TTCGGCCTCAACTTCCTCATCTACCA-3′	5′-TATGACAGTGATCTGGGATGAGTCT-3′	CHIP assay (−99817 to -99566)
*PGC-1*α	402	5′-GCTTCATGGATGTGCTGGG-3′	5′-AGGTCATGGGCTCTACTTTC-3′	CHIP assay (−2390 to -1990)
*PGC-1*α	243	5′-GCAGAGGGCTGCCTTGGAGTG-3′	5′-ATCCAGCTCCCGAATGACGCCAGTC-3′	CHIP assay (−103 to +141)
*PGC-1*α	3251	5′-GTCCCTGCACATGGCTTAT-3′	5′-CAATCCACTCTGACACACA-3′	Promoter cloning (−3170 to +101)
*FoxO6*	3276	5′-CCAGAGGAAGATAAGGATTAT-3′	5′-GATGAGGTCGGCGTAGGAAAG-3′	Promoter cloning (−2977 to +299)
*FoxO6*	792	5′-CCAAGCTTGGGATGTGGGCGGCCA-	5′-GGGATCCCGGCCCGGCTGCTGCGG-	Cloning of FoxO6 (229-492)-His
		GCCCGGC-3′	CCGC-3′	

### EMSA (Electrophoretic mobility-shift assay)

The detailed protocol of EMSA has been described previously [22,25]. Briefly, ^32^P-labelled oligonucleotide corresponding to the putative FoxO6-targeted site (region) in *PGC-1*α promoter was made by polynucleotide kinase (PNK, New England Biolabs) mediated end-labelling reaction in the presence of 50 μCi γ-^32^P-ATP.^32^P-labelled probe was further purified by adsorbing onto DAEA cellulose filter paper (DE-81,Whatman) and eluted out with Tris buffer containing 1 M LiCl after extensive wash in 70% ethanol. Bacteria expressed proteins (0.5 μg) were used to bind ^32^P-labeled oligonucleotide probe in 25 μl binding buffer (25 mM Hepes at pH 7.4, 5 mM MgCl_2_, 4 mM EDTA, 2 mM DTT, 110 mM NaCl, 5 ug/ml BSA and 0.8% Ficoll) at room temperature for 30 min. Protein and DNA complexes were resolved on a 5% (w/v) acrylamide low ionic gel containing 5% (v/v) glycerol at 4°C for at least 3.5 h. Signals on the gels were viewed by autoradiography.

### DNase I footprinting assay

*Taq* DNA polymerase amplified *PGC-1*α promoter fragment −2310 to −1902 was cloned into the T-tailed EcoRV site of yT&A vector to make the yTA (−2310 to −1902)-construct. For labelling the 5′ end of this fragment, this construct was first digested with EcoRI and then labelled with Klenow reaction in the presence of 50 μCiα-^32^P-ATP. The labelling on the upstream vector end was removed by digestion with KpnI and the probe was further purified with DNA purification spin columns (DF300, Geneaid Biotech). Probe (8×10^3^ cpm) and various amounts (0.25 and 0.5 μg) of MBP (maltose-binding protein) or MBP–FoxO6 were incubated in buffer A (10 mM Tris, pH 8.0, 5 mM MgCl2, 1 mM CaCl2, 2mM dithiothreitol, 50 μg BSA/ml, and 2 μg/ml salmon sperm DNA) at 37°C for 30 min before addition of DNase I (0.01 units) and incubated for another 2 min at room temperature. The reaction was stopped by adding 700 μl Stop solution (645 μl ethanol and 55 μl saturated ammonium acetate) and the mixture was left at −20°C for 30 min to precipitate the DNA. After washing with 75% (v/v) ethanol 1–2 times, the residual ethanol was removed by air-dry. DNA was reconstituted in 6 μl gel-loading solution and left at 75°C for 3 min to increase the solubility. Then, samples were spun at 10000 rpm to collect the solution before loading into 6% (w/v) acrylamide sequencing gel buffered with 0.5× TBE. Sequencing gels were run at 1800 V for 2–3 h until the front dye reached the bottom of the gels and the signals on the gels were viewed with autoradiography.

### Polyclonal antibody generation and Western blot

PET32a vector carrying *FoxO6* coding region corresponding to amino acid 229–492 was transformed into BL-21 strain *Escherichia coli* cells and induced by IPTG (isopropyl β-d-thiogalactoside) to express His-tagged FoxO6 (229–492) protein. Recombinant protein was purified by nickel column (Chelating Sepharose, GE healthcare) and aliquots (100–200 μg) of purified protein were mixed with Fraund's adjuvant before injected subcutaneously into the dorsal flank of rabbits every other weeks for 2 months. Blood was collected by cardiac puncture and allowed to coagulate for at least 3 h before centrifuged at 3000 rpm for 30 min for collecting anti-serum. The animal experiments were performed in accordance with the guidelines of the Experimental Animal Care and Use Committee of the National Central University.

The protocol for Western Blot has been described previously [23]. Briefly, aliquots of total lysate (50 μg) in RIPA buffer (20 mM TRIS, 150 mM NaCl, 1% (w/v) deoxycholate, 0.1% (w/v) SDS, 1% (v/v) Triton-X 100, pH 7.8) supplemented with protease inhibitors were run on SDS/PAGE (10% gel) before blotted onto a PVDF membrane (Pall FluoroTrans W membrane, PALL). The PVDF membranes were extensively washed with 1× PBST (PBS containing 0.5% (v/v) Tween 20) before blocked by 5% (w/v) non-fat dried skimmed milk powder in PBST. Primary antibody was diluted 1:1000 in blocking solution (5% (w/v) non-fat dried skimmed milk powder in PBST) and incubated with the blot at 4°C for overnight. After several washes with PBST, HRP (horseradish peroxidase)-conjugated secondary antibody (1:1000 dilution) was added and incubated at room temperature for 1 h. The signals were detected by a chemiluminescence kit (Amersham Pharmacia Biotech) and visualized on X-ray films (Super RX, Fuji Medical X-film; Tokyo, Japan). For detection of Gapdh as internal control, all the blots were stripped, washed, and then incubated with Gapdh antibody (diluted 1:500; Sc-815, Santa Cruz) and HRP conjugated secondary antibody described as above. The antibodies for FoxO1 and PGC-1α were purchased from Cell Signaling (no. 28805) and ABCam (no. ab72230), respectively.

### Oxidative low-intensity exercise and CHIP (chromatin immunoprecipitation) assay

Male mice of 4–6 months old were mounted to a home-made running wheel (circumference: 70 cm) rotating at 7.5 circles/min and the running was allowed to last for an hour (total running length: 315 m). This oxidative low-intensity running was repeated once per day for consecutive 7 days. At the end of last running, mice were sacrificed 2–3 h after exercise when the expression of *PGC-1*α is at its peak level and to avoid the immediate acute effect of the exercise [26]. All animal experiments were performed in accordance with the guidelines of the Experimental Animal Care and Use Committee of the National Central University.

The detailed protocol of CHIP assay has been described before [27]. Briefly, GC (gastrocnemius) and soleus muscles were isolated, quickly minced in cold PBS, and fixed in formaldehyde (1%, v/v) for 20 min. Then, tissues were washed and quenched with cold PBS containing 125 mM glycine before resuspended in lysis buffer (50 mM Tris/HCl, 150 mM NaCl, pH 8.1, 0.1% (w/v) SDS, 1% (w/v) Triton X-100) and sonicated with at least five cycles of 30 s 140–150 W pulse. The lysate was cleared by spinning and then protein A/G agarose (80 μl) was added to the supernatant and incubated at 4°C for 1 h before spun at 4000 rev./min for 5–10 min at 4°C to clear the solution. The supernatant was transferred to a new tube and 1 μg specific antibody was added. The binding was allowed to proceed at 4°C overnight. Protein A/G agarose (80 μl) was added and incubated for another hour to capture the immune complex. The beads were collected and washed four times in lysis buffer for 10 min; then, washed once in LiCl buffer and twice in 1× TE for 5 min. The immune complex was eluted twice with 250 μl of elution buffer and the eluate was heated at 65°C for at least 6 h to reverse the cross-linking. DNA was extracted with phenol/chloroform twice and further purified using a PCR purification Kit (Geneaid; Taipei, Taiwan) before amplified with primers targeting *PGC-1*α promoters.PCR of a *PGC-1*α upstream site (−99857 to −99642) served as a negative control. The relative amount of co-immunoprecipitated DNA was determined by PCR and compared with that of input DNA.

## RESULTS

### FoxO6 is ubiquitously expressed but enriched in oxidative tissues

To explore the function of FoxO6 in the adult tissues, we firstly examined the expression of *FoxO6* mRNA in various tissues of adult mice by qRT-PCR. Similar to previous studies [14,28], we found that *FoxO6* is highly expressed in the brain tissues, with the highest level in the pituitary, implying that FoxO6 might be involved in the regulation of endocrine system. The expression of *FoxO6* in the heart, liver and kidney is rather low; on the contrary, substantial amount of it was expressed in the skeletal muscles, especially higher in the oxidative type I soleus muscle, suggesting that *FoxO6* expression is preferentially enriched in tissues relying on oxidative metabolism as their major energy source.

**Figure 1:**
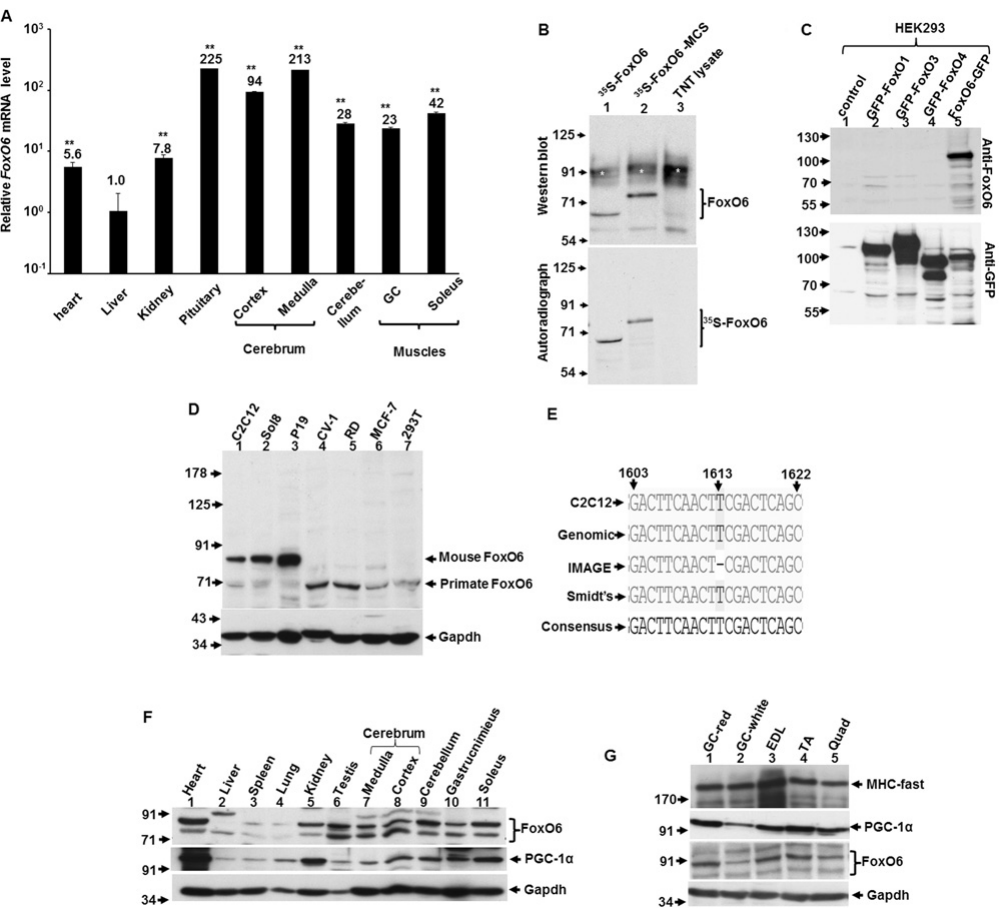
Detecting the expression of *FoxO6* at both mRNA and protein levels in various tissues (**A**) The expression of *FoxO6* mRNA in various mouse tissues (*N*=3) was determined by qRT-PCR. The expression level of *FoxO6* in liver was arbitrarily set as 1-fold and compared by the levels in other tissues.**, *P*<0.01 as compared with that of liver. (**B**) *In vitro* translated ^35^S-FoxO6 was detected with either auto-radiography (lower panel) or Western blot (upper panel) using home-made polyclonal antibody. FoxO6-MCS is a chimaeric protein containing *FoxO6* CDS and the multiple cloning sites (from BamHI to XbaI) of pCDNA3 vector. Lysate: reticulocyte total lysate used for translation. *, non-specific band caused by haemoglobin in the reticulocyte. (**C**) GFP fused FoxOs were expressed in HEK-293 cells and their expression were detected by GFP antibody (lower panel) or FoxO6 antibody (upper panel). Total lysate (50 μg) from mouse and primate cells (**D**) or various mouse tissues (**F** and **G**) were examined for their expression of FoxO6 by Western blot. The signals of Gapdh serve as input control. (**E**) Alignment of FoxO6 cDNA sequences (1603–1622) reported by IMAGE Consortium, Smidt's group, and our group (C2C12). The corresponding genomic sequence is also aligned. Numbering is according to Smidt's clone. GC, gastrocnemius; EDL, extensor digitorum longus; TA, tibialis anterior; Quad, quadriceps.

As the study of *FoxO6* had been hindered by the lack of commercially available FoxO6-specific antibody, we set out to generate FoxO6-specific antibody by injecting recombinant FoxO6 protein fragment (Supplementary Figure S1, available at http://www.bioscirep.org/bsr/033/bsr033e045add.htm) corresponding to the least conserved domain (amino acid 229–492, Supplementary Figure S2, available at http://www.bioscirep.org/bsr/033/bsr033e045add.htm) among FoxOs into rabbit to induce polyclonal antibody. This polyclonal antibody recognizes *in vitro* translated FoxO6 protein and GFP–FoxO6 transiently expressed in HEK-293T (HEK-293 cells expressing the large T-antigen of SV40) cells without cross-reacting with other FoxOs (Figures 1B and 1C and Supplementary Figure S3, available at http://www.bioscirep.org/bsr/033/bsr033e045add.htm), demonstrating that it is a useful tool for studying FoxO6-specific function and expression level. Using this antibody, we found that the molecular weight of endogenous murine and primate FoxO6 is about 84 and 70 kDa, respectively. As the formula molecular weight of reported murine FoxO6 (559 amino acids; accession no. : NM_194060/XM_906986) is about 58 Kd and much higher molecular weights were found in *in vitro* translated FoxO6 protein (68 kDa; Figure 1B, lane 1) and in endogenous FoxO6 proteins, it suggests that FoxO6 protein is significantly modified after translation in mouse cells and a longer form of *FoxO6* isoform is expressed *in vivo*. Actually, a FoxO6 transcript (accession no. : BC086628) with 640 amino acids has been reported by the IMAGE Consortium, in which the T nucleotide at 1613 is deleted resulting in a reading frame shift in the C-terminal. However, we found that the FoxO6 transcript in C2C12 myoblasts did contain the 1613 T nucleotide (Figure 1E) and its sequence is the same as that reported by Smidt's group [14], suggesting that a longer isoform with extended 5′-end might be the major transcript expressed in mouse cells. To date, human *FoxO6* has not been cloned and the lower molecular weight of green monkey (CV-1) and human (MCF-7, RD and HEK-293) FoxO6 implies that, during primate evolution, *FoxO6* gene might either be truncated or mutated at amino acids that are critical targets of post-translational modifications.

In adult mouse tissues the level of FoxO6 protein correlated well with that of mRNA, and it was also highly expressed in the brain tissues and the oxidative soleus muscle (Figures 1F and 1G). In skeletal muscles, the lowest levels of both PGC-1α and FoxO6 were found in the white portion of the GC muscle (Figure 1G). Surprisingly, high level of FoxO6 protein was observed in the heart and kidney where low level of *FoxO6* mRNA was detected, implying that in these tissues either the translation efficiency or the protein stability of *FoxO6* was highly increased by unknown mechanisms. As the expression pattern of FoxO6 protein correlates well with that of PGC-1α, it suggests that the expression of *FoxO6* might be targeted by PGC-1α, a master regulator of oxidative metabolism.

### PGC-1α enhances *FoxO6* expression

**Figure 2:**
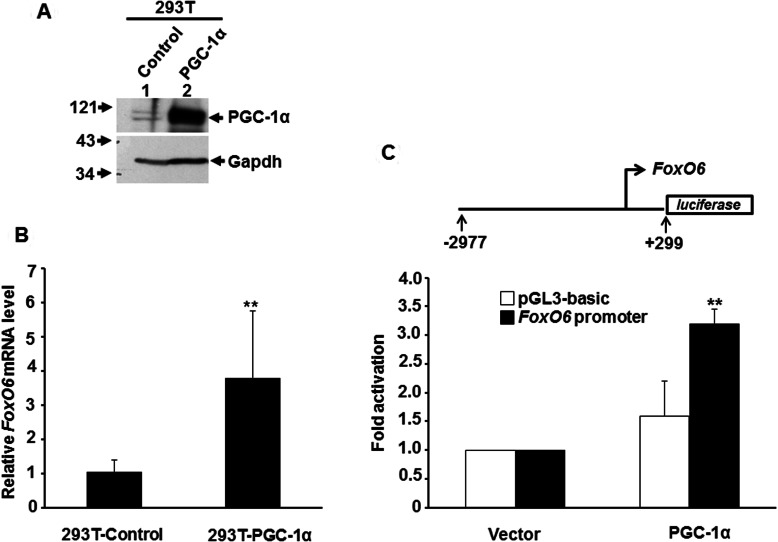
PGC-1αactivates *FoxO6* expression 293T fibroblasts were stably transfected with either pYCAGIP-*PGC-1**α*** or control vector to generate stable clones. The expression of *PGC-1**α*** in both clones was examined by Western blot (**A**), and their expression level of *FoxO6* was examined by qRT-PCR (**B**). The level of FoxO6 in control cells was arbitrarily set as 1-fold.**, *P*<0.01 as compared with that of control cells. (**C**) FoxO6 promoter (−2977 to +299) driven luciferase reporter was co-transfected with/without *PGC-1**α*** expression vector into C2C12 myotubes and harvested 72 h after transfection. The promoter activity at the absence of *PGC-1**α*** was arbitrarily set as 1-fold. **, *P*<0.01 as compared with that of vector control.

The regulation of *FoxO6* by PGC-1α was examined by over-expressing PGC-1α in HEK-293T cells, which allowed super-transfection of pPyCAG-IP-PGC-1α vector due to large T antigen driven replication of this vector. Using qRT-PCR, we found that *FoxO6* mRNA level was significantly increased by *PGC-1*α overexpression (Figures 2A and 2B), suggesting that *FoxO6* is a *bona fide* target of PGC-1α. It was of interest to know whether PGC-1α directly co-activated the promoter activity of *FoxO6*. To address this issue, we cloned the promoter of *FoxO6* and co-transfected it with *PGC-1*α into C2C12 cells induced to differentiate. We found that *FoxO6* promoter activity was significantly enhanced by PGC-1α in differentiated myotubes, demonstrating that PGC-1α directly targets *FoxO6* promoter and promotes its expression in oxidative tissues.

### FoxO6 represses PGC-1α expression in myogenic cells

**Figure 3:**
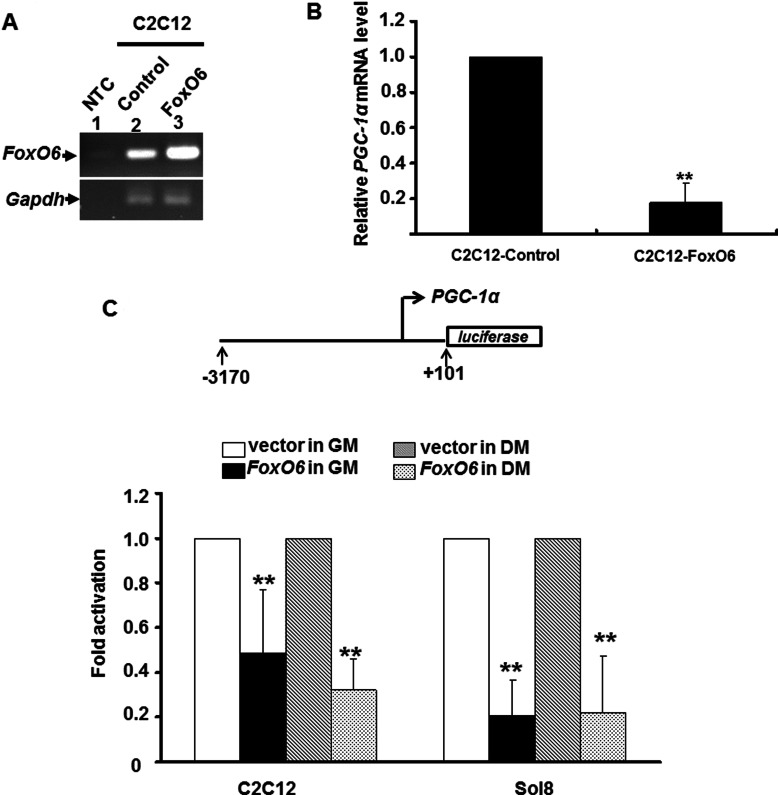
FoxO6 represses *PGC-1*α expression in myogenic cells FoxO6 was stably expressed in C2C12 cells and their *FoxO6* expression levels were examined by RT-PCR (**A**). The expression levels of *PGC-1*αin control and *FoxO6* over-expressed C2C12 myoblasts were determined with qRT-PCR (**B**). The expression level of *PGC-1*αin control cells was arbitrarily set as 1. **, *P*<0.01 as compared with that of control cells. NTC, no template control (**C**). *PGC-1*αpromoter (−3229 to +22) driven luciferase reporter was co-transfected with *FoxO6* expression vector into C2C12 and Sol8 cells and then incubated in either GM or DM for 48 h before harvested. The promoter activity in the absence of *FoxO6* was arbitrarily set as 1-fold. **, *P*<0.01 as compared with vector control.

It has been shown that FoxO1 activate *PGC-1*α expression in hepatocyte to enhance gluconeogenesis [17], butin muscle it down-regulates type I fibre and oxidative genes [12], suggesting that FoxOs may regulate PGC-1α in a tissue-specific manner. Whether the same relationship exists between *FoxO6* and *PGC-1*α needs to be examined. We found that overexpression of *FoxO6*, suppressed *PGC-1*α expression in confluent myoblasts kept in GM (Figure 3B), indicating that FoxO6 is a repressor of *PGC-1*α expression in skeletal muscle.

It is of interest to know whether FoxO6 directly regulates the expression of *PGC-1*α in muscle cells. To address this issue, *PGC-1*α promoter (−3170 to +101) was co-transfected with *FoxO6* into C2C12 cells to reveal if it is targeted by FoxO6. As in the stable clones, FoxO6 repressed *PGC-1*α promoter activity regardless of the differentiation condition (Figure 3C), suggesting that FoxO6 directly targets *PGC-1*α promoter

### FoxO6 targets an upstream region in *PGC-1*α promoter

To further define the mechanism by which FoxO6 regulate *PGC-1*α promoter, we set out to make series of 5′-end deletion mutants of *PGC-1*α promoter to identify the response elements targeted by FoxO6. Deletion of the region −3170 to −2224 had no effect on the FoxO6-mediated repression (Figures 3A and 3B). However, further deletion to −763 abolished the repression (Figures 3A and 3B), implying that the region −2223 to −763 contains FoxO6-targetd sites, and this speculation was confirmed by the strong repression of the region −2252 to −710 by FoxO6. Further dissection of this region showed that the significant repression was still preserved within the region −2252 to −1843; indicating that this region harbours major FoxO6 target sites. Therefore this region was further dissected into four fragments and examined for their repression by FoxO6. Among these four fragments, only the activity of the fragment −2252 to −2156 was repressed by FoxO6 (Figures 3C and 3D), as a result, we focused our further study on this region.

### FoxO6 directly binds to an upstream A/T-rich element in the *PGC-1*α promoter

**Figure 4:**
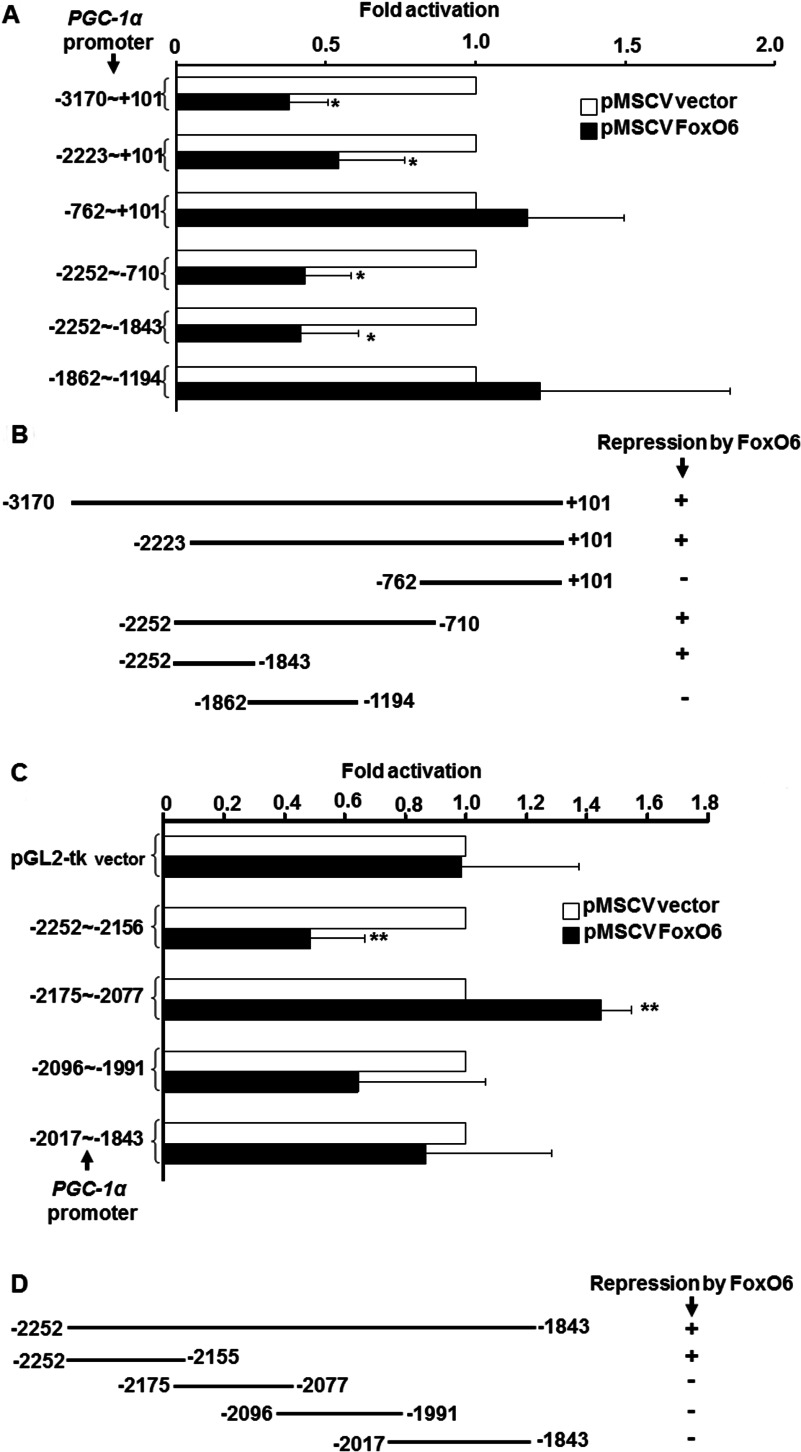
FoxO6 targets an upstream region of the *PGC-1*α promoter In (**A**) and (**C**), the activity of full length and deletion mutants of *PGC-1*α promoter by FoxO6 was determined by transient transfection assays in C2C12 myoblasts kept in GM as described in Figure 3. All numbering of promoter regions are relative to the transcription initiation site. For promoter regions without containing the proximal region, a TK (thymidine kinase) minimal promoter was inserted downstream to drive the basal expression. Schematics in (**B**) and (**D**) summarized the regulation of these promoter regions by FoxO6. The promoter activity in the presence of pMSCV vector, but absence of pMSCV–FoxO6 was set as 1-fold activation. The significance of FoxO6-mediated repression was examined by student's *t* test. Results shown are means and S.D. of at least three independent experiments. * and **, *P*<0.05 and <0.01, respectively, as compared with that of pMSCV vector.

**Figure 5:**
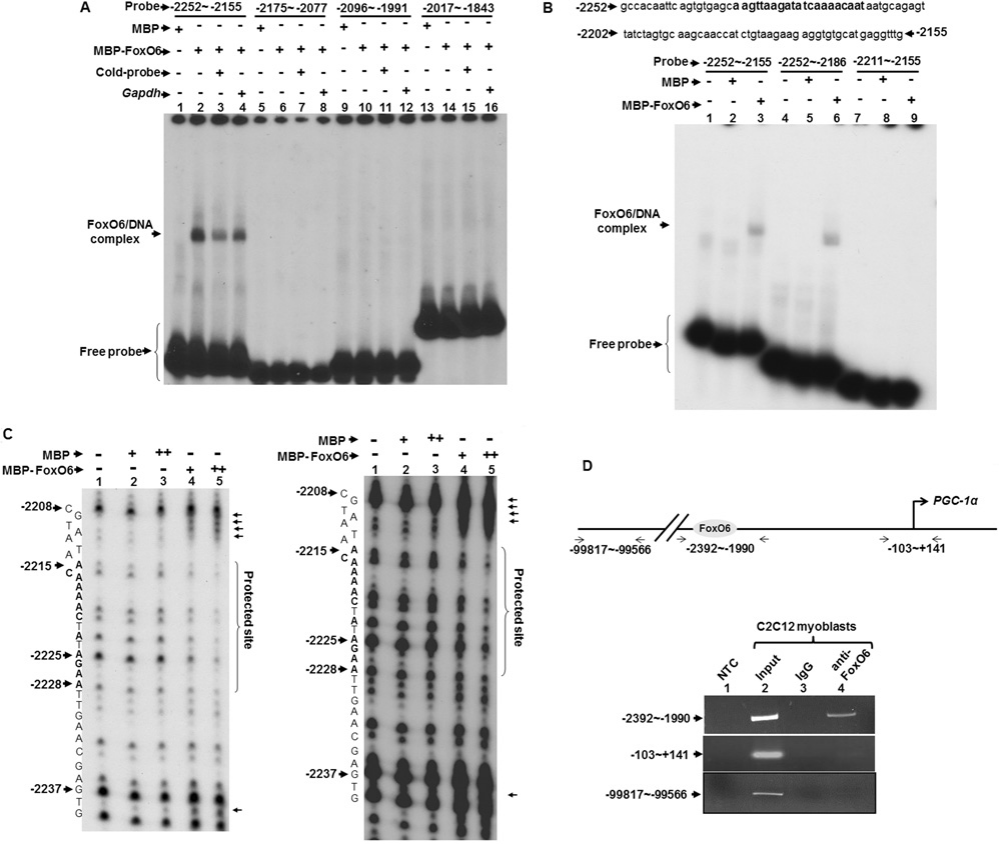
FoxO6 directly binds to an upstream A/T-rich element in the *PGC-1*α promoter (**A** and **B**) Bacteria expressed MBP or MBP-FoxO6 (0.5 μg) was used to bind ^32^P-labelled DNA probes spanning the region −2252 to −1843 in EMSA. Cold probe and *Gapdh* DNA (×100) were included in the reaction to test the binding specificity of MBP-FoxO6 to each probe. (**C**) DNase I footprinting of MBP-FoxO6-binding sites in *PGC-1*α promoter. ^32^P-labelled DNA probes (−2252 to 1843) was digested with DNAse I in the presence and absence of MBP or MBP-FoxO6 proteins before resolved on a 6% sequencing gel. The signals on the gel were viewed by autoradiography. The corresponding position of each base in the probe is shown to the left. The images of both short (16 h, left panel) and long (48 h, right panel) exposures of a representative gel are shown here. (**D**) CHIP assay using antibodies against FoxO6 or non-specific IgG. Precipitated chromatin was amplified by primer sets targeting different regions of the *PGC-1*α promoter. The priming sites are shown on the top panel. NTC, no template control for PCR. The upstream priming sites (−99875 to −99642) were used as a negative control of CHIP assay.

Although FoxO6 can repress the activity of the region −2252 to −2156, it does not necessarily mean that FoxO6 binds directly to this region. Apart from the direct binding, FoxO6 can also repress *PGC-1*α transcription either by blocking the activity of other DNA-binding transcription factors through protein–protein interaction or by regulating the expression of other upstream transcription factors. To further define the mechanisms by which FoxO6 repressed *PGC-1*α promoter activity, we examined the direct binding of FoxO6 to *PGC-1*α promoter DNA with electrophoresis gel mobility shift assay (EMSA). The binding of MBP–FoxO6 to the four fragments spanning the region −2252 to −1843 was examined and specific binding was only found to fragment −2252 to −2155 (Figure 4 A), confirming the results observed in the transfection assay (Figures 3C and 3D). Deletion of the region −2185 to −2155 had no effect on the binding (Figure 4B, lanes 4–6), but deletion of the region −2252 to −2212 abrogated the binding (Figure 4B, lanes 7–9). Therefore the binding sites of FoxO6 should localize in the region −2252 to −2186. Since the consensus sequence: (**TTGTTTAC)** of the FoxO-binding sites was a short A/T rich element, we examined whether a similar sequence existing within the region −2252 to −2186. As expected, an A/T rich region (−2228 to −2211) was found in the middle of this region. To further verify if FoxO6 did bind to this A/T-rich region, a DNAse I footprinting assay was performed and we found that FoxO6 specifically protected the region −2228 to −2215, especially on nucleotides A in this region (Figure 5C). Enhanced digestion of two neighbouring regions (−2238 and −2212 to −2209) was also observed upon FoxO6 binding, implying that this binding had induced dramatic conformational change in the DNA. The binding of FoxO6 to this region *in vivo* was confirmed using CHIP assay, in which we found that FoxO6 specifically targeted this region without binding to the proximal promoter region (Figure 5D). These observations suggest that FoxO6 directly binds to the A/T-rich element (AAGATATCAAAACA, −2228 to −2215) to repress *PGC-1*α transcription. It will be interesting to know how FoxO6 and other factors binding to this distal upstream *cis*-element communicate with polymerase II machinery to compromise its transcriptional activation.

### Oxidative low-intensity exercise relieves FoxO6 mediated repression of *PGC-1*α

**Figure 6:**
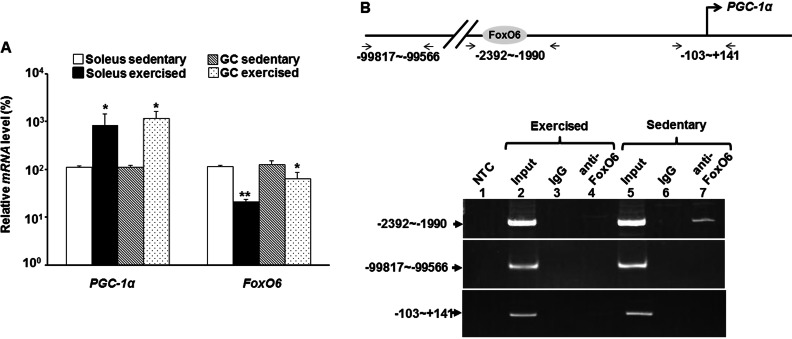
Exercise releases FoxO6-mediated *PGC-1*α repression (**A**) The expression levels of *PGC-1*α and *FoxO6* in hind leg muscles of sedentary and exercised mice (*N*=3) were determined with qRT-PCR. The expression level of each gene in the sedentary mice was arbitrarily set as 100%.* and **, *P*<0.05 and *P*<0.01, respectively, as compared with that of the same muscle in sedentary mouse. (**B**) Binding of FoxO6 to *PGC-1*α promoter in hind leg muscles was examined with CHIP assay as described in Figure 5.

The enriched expression level in oxidative muscle and direct repression of *PGC-1***α** expression presents a conundrum for FoxO6 in metabolic regulation. To better understand its role in oxidative metabolism regulation, the effect of oxidative exercise on the expression of *PGC-1***α** and *Foxo6* was examined. *PGC-1*α expression was highly induced upon exercise in both GC and soleus muscles (Figure 6A). However, the expression of *FoxO6* was repressed by oxidative exercise in both muscles, suggesting that FoxO6-mediated *PGC-1***α** repression can be relieved by exercise (Figure 6A). To confirm this speculation, we examined the binding of FoxO6 to *PGC-1*α promoter *in vivo* by CHIP assay. As expected, FoxO6 bound to *PCG-1***α** promoter strongly in sedentary muscles but was released from the promoter in exercised muscles (Figure 6B) to allow the activation of *PGC-1*α promoter in exercised muscles.

## DISCUSSION

### Sequence comparison of FoxO6-binding sites

Although FoxO6 has been identified for a few years, very few target genes have been identified until recent years, partially due to its unique distribution and lack of available antibody. It is conceivable that FoxO6 should play critical roles in the development of nervous system due to its enriched expression in the central nervous system. A FoxO–Pak1 transcriptional loop has now been identified to regulate neuronal polarity during early embryogenesis [29]. Reduced expression of FoxO6 and other members of the family in the cerebellar granule neurons abrogate their axo-dendritic polarity. This polarity can be rescued by overexpression of *Pak1*, demonstrating its role downstream of FoxO6. Direct targeting of FoxO6 on the *Pak1* promoter to activate its expression in the neurons to regulate their axo-dendritic polarity is also demonstrated. The FoxO6-binding site (TGTTTAAACG) is located in the −1223 to −1212 region of the rat *Pak1* promoter and complies with the rules of A/T rich and distal to the transcriptional initiation site. Like other FoxOs, FoxO6 has been implicated in the hepatic gluconeogenesis upon fasting by activating the expression of gluconeogenic enzymes, such as *PPECK* and *G6Pase*, and which can be blunted by insulin [28]. The insulin response element in the G6Pase gene promoter consists of 3 T/A-rich heptamers, but whether they are directly bound by FoxO6 remains to be demonstrated.

**Figure 7:**
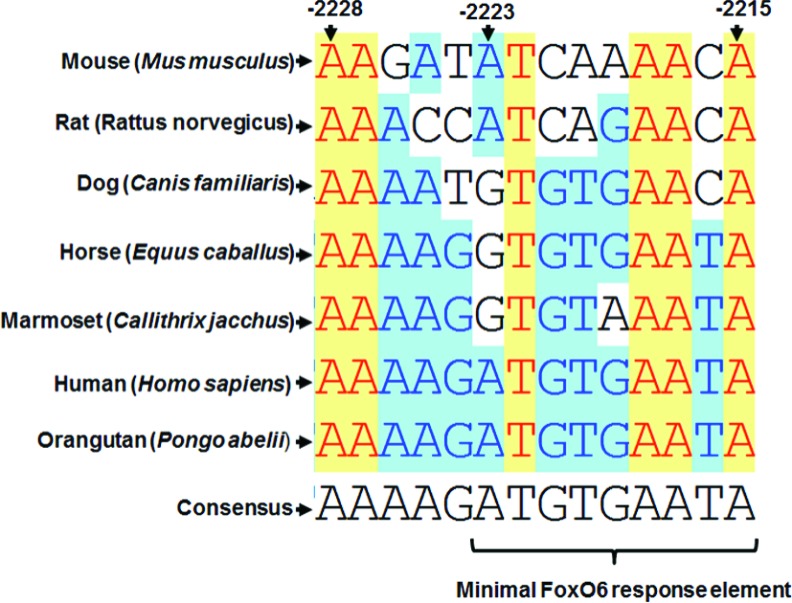
FoxO6-targeted site in the *PGC-1*α promoter is conserved among mammals *PGC-1*α promoters from various mammals were aligned and compared with Vector NTI software and the alignment of the region corresponding to the FoxO6-targeted site in mouse *PGC-1*α is summarized here. Numbering on the top represents the location in mouse *PGC-1*α promoter relative to the major transcription initiation site. The putative minimal FoxO6 response element (−2223 to −2215) is indicated at the bottom.

The FoxO6-protected site (AAGATATCAAAACA, −2228 to −2215) in the *PGC-1*α promoter complies with the same A/T-rich rule and is the third FoxO6 target site identified to date. Since the FxO6 target site in the *Pak1* promoter is also bound by other FoxOs, it will be interesting to know whether the same scenario happens to that in the *PGC-1*α promoter. Alignment of the *PGC-1*α promoter sequences from several mammals (Figure 7A) shows that the FoxO6-binding site in *PGC-1*α promoter is only marginally conserved (43% identity). However, some of the A/T bases are 100% conserved among mammals, suggesting their importance in the transcriptional regulation of *PGC-1*α gene by FoxO6. The divergence of other bases may reflect differential regulation of *PGC-1*α during evolution of mammals.

Although FoxO6 protected the region −2228 to −2215, we also found that the promoter region −2223 to +101 could be well repressed by FoxO6 (Figures 4A and 4B), suggesting that the region −2223 to −2215 (ATCAAAACA) can serve as a minimal FoxO6 response element. We are currently demonstrating that whether this element can confer target promoters negative regulation by FoxO6.

### *PGC-1*α and *FoxO6* form a regulatory loop for oxidative metabolism

Multiple pathways have been found to activate PGC-1α expression, including Mef2-dependent auto-regulation [30], β-adregenic signalling pathway mediated muscle contraction nerve signals [31,32] and hormones, such as thyroid hormone or inhibiting, such as insulin, activating oxidative metabolism [17,33,34]. Insulin represses PGC-1α expression in hepatocyte and its signal is mediated by the PI3K–Akt pathway to phosphrylate members of the FoxO family of transcription factors. Phosphorylated FoxOs are shuttled out of the nucleus, so that they cannot bind to the IRES (insulin response elements) on the *PGC-1*α promoter to activate its expression [17]. In muscle, we previously have found that MRFs (myogenic regulatory factors), especially MyoD and Myogenin, can strongly activate *PGC-1*α expression [35]. As both MRFs and Mef2 are essential for muscle formation/differentiation and they are abundant in differentiating myotubes, it will be interesting to know how these transcription factors communicate with FoxO6 on the *PGC-1*α promoter to determine its expression level and the oxidative metabolism status of muscles in resting and exercising subjects.

**Figure 8:**
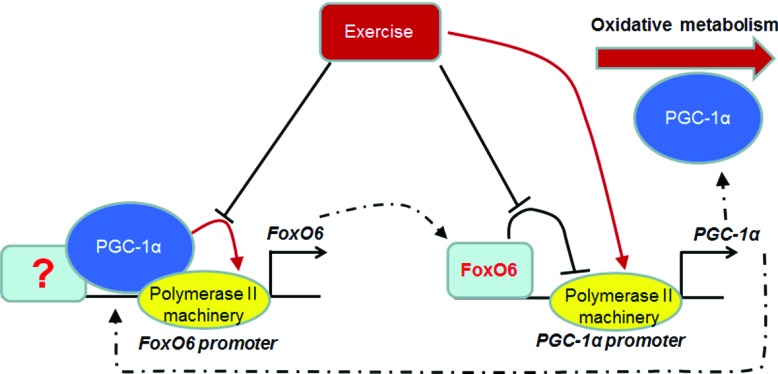
FoxO6 and PGC-1α form a regulatory loop PGC-1α is recruited by unknown transcription factors to co-activate *FoxO6* promoter activity, but FoxO6 directly binds to *PGC-1*α promoter to repress its activity. However, both events can be ameliorated by exercise that further induces *PGC-1*α expression to promote oxidative metabolism. Dashed lines indicate movement of transcription factors.

The relief of FoxO6-mediated *PGC-1*α repression by oxidative low-intensity exercise has opened a new window for understanding the balance of oxidative and glycolytic metabolism in muscle. The higher expression level in oxidative muscle and its repressive effect on *PGC-1*α promoter activity has puzzled us until we found that exercise can release the repression, especially in soleus muscle. These observations suggest PGC-1α and FoxO6 form a regulative loop to maintain the expression level of each other and this balance can be tipped by factors or messengers, such as Mef2 and Ca^+2^, mediating signalling pathway of exercise [36] to determine the final level of oxidative respiration in the cells (Figure 8). The higher FoxO6 levels in oxidative tissues may reflect its transcriptional activation by PGC-1α before the amount of FoxO6 is high enough to repress PGC-1α expression, implying that PGC-1α promoter may have lower sensitivity to FoxO6 repression, so it can achieve higher PGC-1α levels in oxidative tissues, which also allows higher level of FoxO6 to accumulate. It will be interesting to know how intracellular exercise signalling pathways relieve the repression of *PGC-1*α by FoxO6 and whether these mechanisms can reset the sensitivity of *PGC-1*α promoter to FoxO6-mediated repression.

Although FoxO1 has been found to activate *PGC-1*α expression in hepatocytes [17], the same regulation has not been observed in skeletal muscle. On the contrary, we found that both FoxO1 and FoxO6 actively repress *PGC-1*α promoter activity in myoblast cells (Figure 3 and results not shown), this functional discrepancy of FoxOs in these two organs might reflect different roles of these two organs in metabolism: as liver is a source and muscle is a sink of glucose. Both FoxO1 and FoxO6 have been found to increase gluconeogenesis in liver to increase blood glucose level during fast [28]. However, overexpression of FoxOs in muscle reduces type I fibres [12] and induces atrophy by activating the expression of muscle-specific ubiquitin E3 ligases Atrogin-1 and MuRF-1 [37,38]. Furthermore, this atrophic effect can be rescued by *PGC-1*α overexpression. Therefore these data suggest that FoxOs and PGC-1α antagonize each other's activity to maintain the homeostasis of metabolism in muscle. As overexpression of *PGC-1*α strongly promotes oxidative respiration and inhibits catabolism, compromising of its activity is important for the normal muscular composition, in which both fast and slow fibre are required and proper fibre type composition and muscle/fibre size are arranged to meet the demand of either fast or slow movements. Therefore the antagonism of *PGC-1*α promoter activity by FxO6 and its relief under certain physiological condition represents a novel mechanism regulating normal muscle metabolism and fibre type composition.

### Repression of *PGC-1*α by FoxO6 may be involved in the pathogenesis of degenerated diseases

In obesity and T2D (type 2 diabetes) patients, their skeletal muscle shows a phenotype characteristic of reduced oxidative enzyme activity, increased glycolytic activity and increased lipid content. Reduced *PGC-1*α expression is observed in some subjects of both diseases [39,40], demonstrating its importance in muscular metabolism. The key glucose transporter, Glut4, on the plasma membrane is also reduced in T2D patients and the expression of *Glut4* in cultured skeletal muscle cells can be restored by *PGC-1α overexpression* through a Mef2-dependent pathway [41]. Therefore restoring the expression of *PGC-1*αto normal level seems to be a priority in the management of these diseases. Recent studies have shown that *PGC-1*α can suppress ROS (reactive oxygen species) and neurodegeneration [42]; furthermore, its expression in degenerated neuron diseases, such as Alzheimer disease, is significantly reduced [43,44]. Considering the importance of preventing the initiation of these degenerated diseases, it is of interest to know whether the reduced expression of *PGC-1*α in these subjects affected by DM or degenerated neural diseases is caused by the repressive activity of FoxO6 as discovered in this study. This notion has been supported by the observation that reduction of *FoxO6* expression level by infusion of shRNA (small-hairpin RNA)-expressing virus shows significant beneficial effect on db/db mice [28]. Therefore, it is important to characterize the expression pattern of *PGC-1*α and *FoxO6* in these diseased subjects and reveal their correlation with the progress of the diseases.

## Online data

Supplementary data

## References

[1] Accili D., Arden K. C. (2004). FoxOs at the crossroads of cellular metabolism, differentiation, and transformation. Cell.

[2] Tran H., Brunet A., Griffith E. C., Greenberg M. E. (2003). The many forks in FOXO's road. Sci. STKE.

[3] Zhao H. H., Herrera R. E., Coronado-Heinsohn E., Yang M. C., Ludes-Meyers J. H., Seybold-Tilson K. J., Nawaz Z., Yee D., Barr F. G., Diab S. G. (2001). Forkhead homologue in rhabdomyosarcoma functions as a bifunctional nuclear receptor-interacting protein with both coactivator and corepressor functions. J. Biol. Chem..

[4] Barthel A., Schmoll D., Unterman T. G. (2005). FoxO proteins in insulin action and metabolism. Trends Endocrinol. Metab..

[5] Nakamura N., Ramaswamy S., Vazquez F., Signoretti S., Loda M., Sellers W. R. (2000). Forkhead transcription factors are critical effectors of cell death and cell cycle arrest downstream of PTEN. Mol. Cell Biol..

[6] Kortylewski M., Feld F., Kruger K. D., Bahrenberg G., Roth R. A., Joost H. G., Heinrich P. C., Behrmann I., Barthel A. (2003). Akt modulates STAT3-mediated gene expression through a FKHR (FOXO1a)-dependent mechanism. J. Biol. Chem..

[7] Burgering B. M., Kops G. J. (2002). Cell cycle and death control: long live Forkheads. Trends Biochem. Sci..

[8] Bois P. R., Grosveld G. C. (2003). FKHR (FOXO1a) is required for myotube fusion of primary mouse myoblasts. EMBO J..

[9] Hribal M. L., Nakae J., Kitamura T., Shutter J. R., Accili D. (2003). Regulation of insulin-like growth factor-dependent myoblast differentiation by Foxo forkhead transcription factors. J. Cell Biol..

[10] Sandri M., Sandri C., Gilbert A., Skurk C., Calabria E., Picard A., Walsh K., Schiaffino S., Lecker S. H., Goldberg A. L. (2004). Foxo transcription factors induce the atrophy-related ubiquitin ligase atrogin-1 and cause skeletal muscle atrophy. Cell.

[11] Stitt T. N., Drujan D., Clarke B. A., Panaro F., Timofeyva Y., Kline W. O., Gonzalez M., Yancopoulos G. D., Glass D. J. (2004). The IGF-1/PI3K/Akt pathway prevents expression of muscle atrophy-induced ubiquitin ligases by inhibiting FOXO transcription factors. Mol. Cell..

[12] Kamei Y., Miura S., Suzuki M., Kai Y., Mizukami J., Taniguchi T., Mochida K., Hata T., Matsuda J., Aburatani H., Nishino I., Ezaki O. (2004). Skeletal muscle FOXO1 (FKHR) transgenic mice have less skeletal muscle mass, down-regulated Type I (slow twitch/red muscle) fiber genes, and impaired glycemic control. J. Biol. Chem..

[13] Hishiya A., Iemura S., Natsume T., Takayama S., Ikeda K., Watanabe K. (2006). A novel ubiquitin-binding protein ZNF216 functioning in muscle atrophy. EMBO J..

[14] Jacobs F. M., van der Heide L. P., Wijchers P. J., Burbach J. P., Hoekman M. F., Smidt M. P. (2003). FoxO6, a novel member of the FoxO class of transcription factors with distinct shuttling dynamics. J. Biol. Chem..

[15] Hoekman M. F., Jacobs F. M., Smidt M. P., Burbach J. P. (2006). Spatial and temporal expression of FoxO transcription factors in the developing and adult murine brain. Gene Exp. Patterns.

[16] van der Heide L. P., Jacobs F. M., Burbach J. P., Hoekman M. F., Smidt M. P. (2005). FoxO6 transcriptional activity is regulated by Thr26 and Ser184, independent of nucleo-cytoplasmic shuttling. Biochem. J..

[17] Daitoku H., Yamagata K., Matsuzaki H., Hatta M., Fukamizu A. (2003). Regulation of PGC-1 promoter activity by protein kinase B and the forkhead transcription factor FKHR. Diabetes.

[18] Wu Z., Puigserver P., Andersson U., Zhang C., Adelmant G., Mootha V., Troy A., Cinti S., Lowell B., Scarpulla R. C., Spiegelman B. M. (1999). Mechanisms controlling mitochondrial biogenesis and respiration through the thermogenic coactivator PGC-1. Cell.

[19] Yoon J. C., Puigserver P., Chen G., Donovan J., Wu Z., Rhee J., Adelmant G., Stafford J., Kahn C. R., Granner D. K. (2001). Control of hepatic gluconeogenesis through the transcriptional coactivator PGC-1. Nature.

[20] Lin J., Wu H., Tarr P. T., Zhang C. Y., Wu Z., Boss O., Michael L. F., Puigserver P., Isotani E., Olson E. N. (2002). Transcriptional co-activator PGC-1 alpha drives the formation of slow-twitch muscle fibres. Nature.

[21] Czubryt M. P., McAnally J., Fishman G. I., Olson E. N. (2003). Regulation of peroxisome proliferator-activated receptor gamma coactivator 1 alpha (PGC-1 alpha) and mitochondrial function by MEF2 and HDAC5. Proc. Natl Acad. Sci. U.S.A..

[22] Hsiao S. P., Huang K. M., Chang H. Y., Chen S. L. (2009). P/CAF rescues the Bhlhe40-mediated repression of MyoD transactivation. Biochem. J..

[23] Lang K. C., Lin I. H., Teng H. F., Huang Y. C., Li C. L., Tang K. T., Chen S. L. (2009). Simultaneous overexpression of Oct4 and Nanog abrogates terminal myogenesis. Am. J. Physiol. Cell Physiol..

[24] Teng H. F., Kuo Y. L., Loo M. R., Li C. L., Chu T. W., Suo H., Liu H. S., Lin K. H., Chen S. L. (2010). Valproic acid enhances Oct4 promoter activity in myogenic cells. J. Cell Biochem..

[25] Hsiao S. P., Chen S. L. Myogenic regulatory factors regulate M-cadherin expression by targeting its proximal promoter elements. Biochem. J..

[26] Akimoto T., Pohnert S. C., Li P., Zhang M., Gumbs C., Rosenberg P. B., Williams R. S., Yan Z. (2005). Exercise stimulates Pgc-1alpha transcription in skeletal muscle through activation of the p38 MAPK pathway. J. Biol. Chem..

[27] Hsiao S. P., Chen S. L. (2010). Myogenic regulatory factors regulate M-cadherin expression by targeting its proximal promoter elements. Biochem. J..

[28] Kim D. H., Perdomo G., Zhang T., Slusher S., Lee S., Phillips B. E., Fan Y., Giannoukakis N., Gramignoli R., Strom S. (2011). FoxO6 integrates insulin signaling with gluconeogenesis in the liver. Diabetes.

[29] de la Torre-Ubieta L., Gaudilliere B., Yang Y., Ikeuchi Y., Yamada T., DiBacco S., Stegmuller J., Schuller U., Salih D. A., Rowitch D. A FOXO-Pak1 transcriptional pathway controls neuronal polarity. Genes Dev..

[30] Handschin C., Rhee J., Lin J., Tarr P. T., Spiegelman B. M. (2003). An autoregulatory loop controls peroxisome proliferator-activated receptor gamma coactivator 1alpha expression in muscle. Proc. Natl Acad. Sci. U.S.A..

[31] Boss O., Bachman E., Vidal-Puig A., Zhang C. Y., Peroni O., Lowell B. B. (1999). Role of the beta(3)-adrenergic receptor and/or a putative beta(4)-adrenergic receptor on the expression of uncoupling proteins and peroxisome proliferator-activated receptor-gamma coactivator-1. Biochem. Biophys. Res. Commun..

[32] Gomez-Ambrosi J., Fruhbeck G., Martinez J. A. (2001). Rapid *in vivo* PGC-1 mRNA up-regulation in brown adipose tissue of Wistar rats by a beta(3)-adrenergic agonist and lack of effect of leptin. Mol. Cell Endocrinol..

[33] Feingold K., Kim M. S., Shigenaga J., Moser A., Grunfeld C. (2004). Altered expression of nuclear hormone receptors and coactivators in mouse heart during the acute-phase response. Am. J. Physiol. Endocrinol. Metab..

[34] Weitzel J. M., Radtke C., Seitz H. J. (2001). Two thyroid hormone-mediated gene expression patterns *in vivo* identified by cDNA expression arrays in rat. Nucleic Acids Res..

[35] Chang J. H., Lin K. H., Shih C. H., Chang Y. J., Chi H. C., Chen S. L. (2006). Myogenic basic helix-loop-helix proteins regulate the expression of peroxisomal proliferator activated receptor-gamma coactivator-1alpha. Endocrinology.

[36] Gundersen K. (2011). Excitation-transcription coupling in skeletal muscle: the molecular pathways of exercise. Biol. Rev. Camb. Phil. Soc..

[37] Brault J. J., Jespersen J. G., Goldberg A. L. (2010). Peroxisome proliferator-activated receptor gamma coactivator 1 alpha or 1 beta overexpression inhibits muscle protein degradation, induction of ubiquitin ligases, and disuse atrophy. J. Biol. Chem..

[38] Sandri M., Lin J., Handschin C., Yang W., Arany Z. P., Lecker S. H., Goldberg A. L., Spiegelman B. M. (2006). PGC-1alpha protects skeletal muscle from atrophy by suppressing FoxO3 action and atrophy-specific gene transcription. Proc. Natl Acad. Sci. U.S.A..

[39] Crunkhorn S., Dearie F., Mantzoros C., Gami H., da Silva W. S., Espinoza D., Faucette R., Barry K., Bianco A. C., Patti M. E. (2007). Peroxisome proliferator activator receptor gamma coactivator-1 expression is reduced in obesity: potential pathogenic role of saturated fatty acids and p38 mitogen-activated protein kinase activation. J. Biol. Chem..

[40] Mootha V. K., Lindgren C. M., Eriksson K. F., Subramanian A., Sihag S., Lehar J., Puigserver P., Carlsson E., Ridderstrale M., Laurila E. (2003). PGC-1alpha-responsive genes involved in oxidative phosphorylation are coordinately downregulated in human diabetes. Nat. Genet..

[41] Michael L. F., Wu Z., Cheatham R. B., Puigserver P., Adelmant G., Lehman J. J., Kelly D. P., Spiegelman B. M. (2001). Restoration of insulin-sensitive glucose transporter (GLUT4) gene expression in muscle cells by the transcriptional coactivator PGC-1. Proc. Natl Acad. Sci. U.S.A..

[42] St-Pierre J., Drori S., Uldry M., Silvaggi J. M., Rhee J., Jager S., Handschin C., Zheng K., Lin J., Yang W. (2006). Suppression of reactive oxygen species and neurodegeneration by the PGC-1 transcriptional coactivators. Cell.

[43] Qin W., Haroutunian V., Katsel P., Cardozo C. P., Ho L., Buxbaum J. D., Pasinetti G. M. (2009). PGC-1alpha expression decreases in the Alzheimer disease brain as a function of dementia. Arch. Neurol..

[44] Cui L., Jeong H., Borovecki F., Parkhurst C. N., Tanese N., Krainc D. (2006). Transcriptional repression of PGC-1alpha by mutant huntingtin leads to mitochondrial dysfunction and neurodegeneration. Cell.

