# Supplementary material for: FoxO6 and PGC-1α form a regulatory loop in myogenic cells
Source: Biosci Rep. 2013 Jun 13;33(3):e00045. doi: 10.1042/BSR20130031 (PMC3681252; doi:10.1042/BSR20130031)
Supplement: Supplementary data [file bsr033e045add.pdf]

Biosci. Rep. (2013) / **33** / art:e00045 / doi 10.1042/BSR20130031

OPEN ACCESS

Original Paper

Bioscience Reports

www.bioscirep.org

Bioscience Reports

SUPPLEMENTARY DATA

FoxO6 and PGC-1α form a regulatory loop in myogenic cells

Shih Ying CHUNG*, Wei Chieh HUANG*, Ching Wen SU*, Kuan Wei LEE*, Hsiang Cheng CHI*, Cheng Tao LIN*, Szu-Tah CHEN†, Kai Min HUANG*, Mu Shiun TSAI‡, Hui Peng YU* and Shen Liang CHEN*[1]

*Department of Life Sciences, National Central University, Jhongli 32001, Taiwan, †Division of Endocrinology and Metabolism, Chang Gung Memorial Hospital, Kweishan 33333, Taoyuan, Taiwan, and ‡Department of Pathology and Medical laboratory, Taiwan Landseed Hospital, Pingjen 32449, Taoyuan, Taiwan

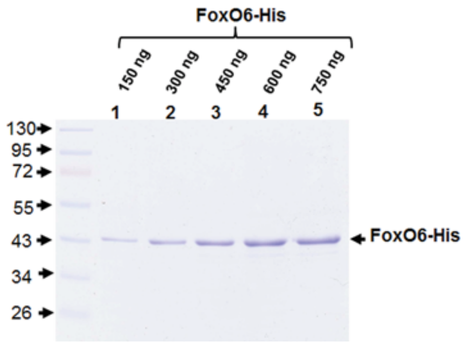

Figure S1 Purified recombinant FoxO6 (amino acid 229–492)-His protein
Various amounts of *E. coli* expressed recombinant protein was purified with nickle-NTA beads and run on 10% SDS/PAGE gels. Protein on the gel was viewed by Coomassie blue staining. Numbers to the left represent molecular weight in kilodalton (kDa).

[1] To whom correspondence should be addressed (email slchen@cc.ncu.edu.tw).

© 2013 The Author(s) This is an Open Access article distributed under the terms of the Creative Commons Attribution Licence (CC-BY) (http://creativecommons.org/licenses/by/3.0/) which permits unrestricted use, distribution and reproduction in any medium, provided the original work is properly cited.

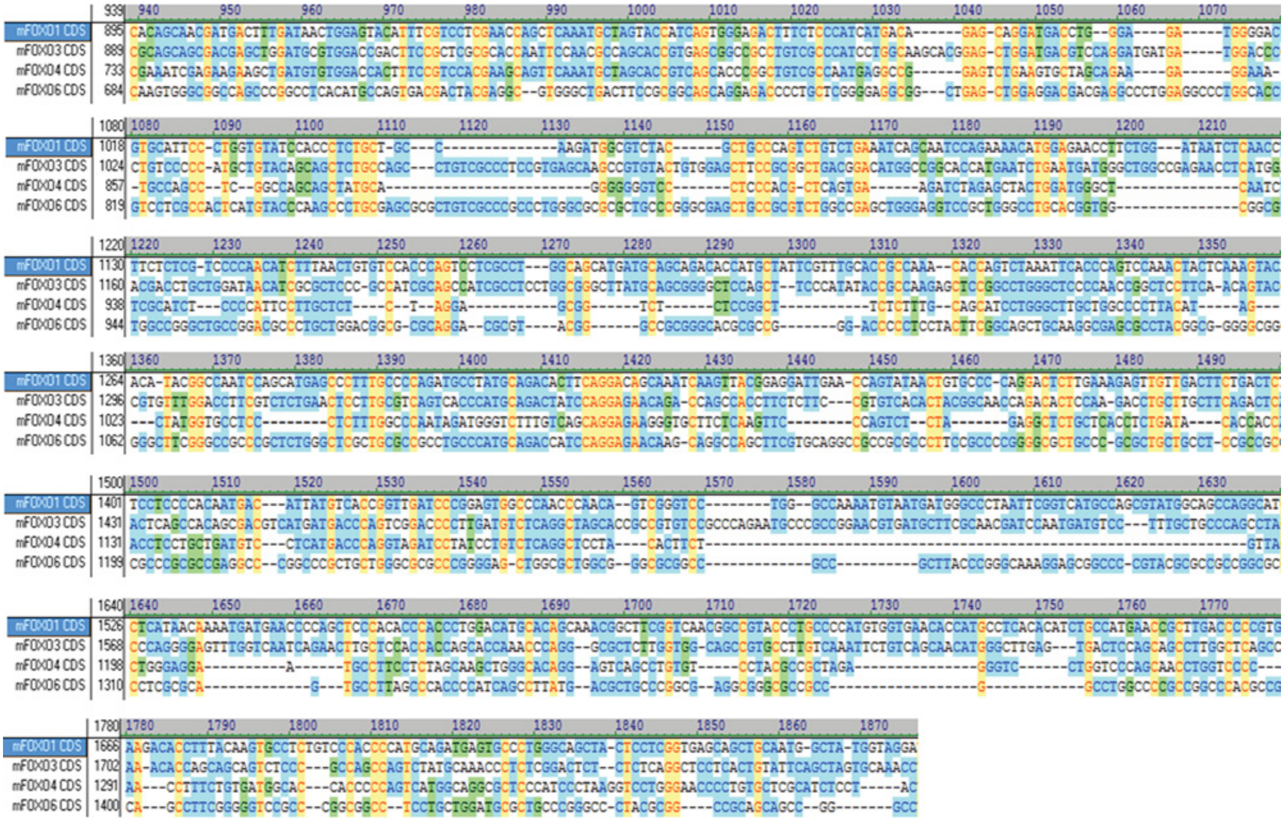

Figure S2 **Alignment of the mouse FoxO family mRNAs**
The sequence of the mouse FoxO family mRNAs was aligned with Vector NTI software and the lease conserved region among FoxO families is shown here.

© 2013 The Author(s) This is an Open Access article distributed under the terms of the Creative Commons Attribution Licence (CC-BY) (http://creativecommons.org/licenses/by/3.0/) which permits unrestricted use, distribution and reproduction in any medium, provided the original work is properly cited.

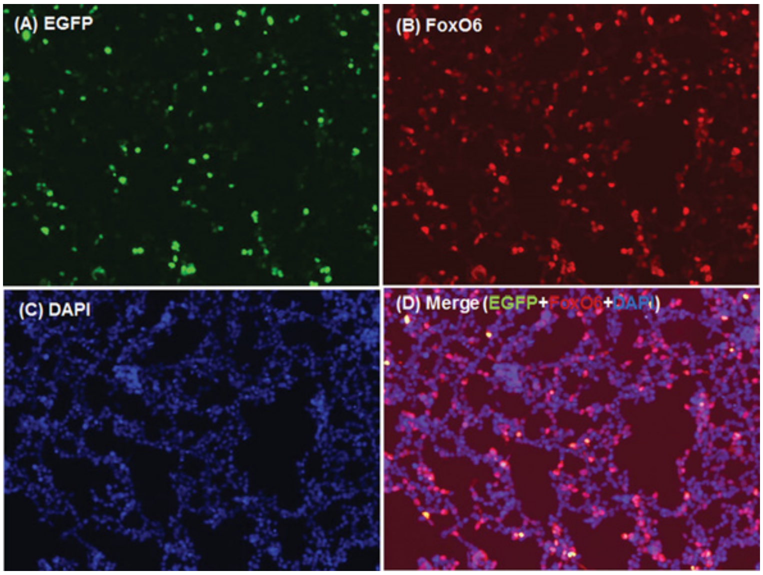

Figure S3 FoxO6 antibody recognizes FoxO6-EGFP in HEK-293 cells
HEK-293 cells were transfected with vector expressing FoxO6-EGFP and the expression of FoxO6-EGFP was recognized by immunofluorescence using FoxO6 antibody and viewed with Texas red-conjugated secondary antibody (**B**).The signals of EGFP (**A**) and DAPI (4′,6-diamidino-2-phenylindole) (**C**) showsignificant overlapping with that of FoxO6 (**D**).

Received 18 March 2013/30 April 2013; accepted 2 May 2013
Published as Immediate Publication 3 May 2013, doi 10.1042/BSR20130031

© 2013 The Author(s) This is an Open Access article distributed under the terms of the Creative Commons Attribution Licence (CC-BY) (http://creativecommons.org/licenses/by/3.0/) which permits unrestricted use, distribution and reproduction in any medium, provided the original work is properly cited.
